# Enhancing the Fluorescence and Antimicrobial Performance of Carbon Dots via Hypochlorite Treatment

**DOI:** 10.3390/nano15030184

**Published:** 2025-01-24

**Authors:** Spyridon Gavalas, Mohammed S. Beg, Ella N. Gibbons, Antonios Kelarakis

**Affiliations:** UCLan Research Centre for Smart Materials, School of Pharmacy and Biomedical Sciences, University of Central Lancashire, Preston PR1 2HE, UK; sgavalas@uclan.ac.uk (S.G.); msbeg@uclan.ac.uk (M.S.B.); egibbons2@uclan.ac.uk (E.N.G.)

**Keywords:** carbon dots, fluorescence, etching, sodium hypochlorite, antifungal

## Abstract

This paper presents a simple, post-synthesis treatment of carbon dots (C-dots) that relies on the oxidizing activity of sodium hypochlorite to induce surface oxidation, etching and pronounced structural rearrangements. The thus treated C-dots (ox-C-dots) exhibit up to six-fold enhancement in quantum yield compared to non-oxidised analogues, while maintaining low levels of cytotoxicity against HeLa and U87 cell lines. In addition, we demonstrate that a range of polymeric materials (polyurethane sponge, polyvinylidene fluoride membrane, polyester fabric) impregnated with ox-C-dots show advanced antifungal properties against *Talaromyces pinophilus*, while their untreated counterparts fail to do so.

## 1. Introduction

Due to their unique fluorescence properties, remarkable resistance to photobleaching and enhanced colloidal stability, carbon dots (C-dots) are ideally suited for applications related to molecular sensing, theragnostics, bioimaging, photothermal and photodynamic therapy, wound healing, antimicrobial treatment, water splitting, photocatalysis, light-emitting diodes, supercapacitors, batteries, anticorrosion, food packaging, nanoforensics and anticounterfeiting [1,2,3,4,5,6,7,8,9]. In addition, C-dots are minimally toxic to the environment and humans, in sharp contrast to heavy metal-based quantum dots [10]. A versatile range of C-dots of varying sizes and graphitisation degrees can be synthesised via cost-effective approaches by exploiting pyrolytic decomposition of precursor materials such as biomass waste and natural products [11,12]. Likewise, C-dots can be generated in situ within polymeric matrices and powder compositions [13,14], following strategies compatible with standard industrial processing. A number of recent reports summarise the current level of understanding of this exciting class of materials and its applications [15,16].

Further, C-dots demonstrate diverse and tuneable photoluminescence (PL) emission modes that can be explained in terms of bandgap transitions of conjugated π-domains within their carbogenic cores, surface defect states on the periphery of the cores that function as capture centres for excitons, embedded organic fluorophores and crosslink enhanced emissions [17,18]. Typically, the strongest PL emissions of C-dots fall within the blue/green region of the spectrum, although intense red/NIR emissive systems have been previously reported for certain systems [19].

Post-synthesis surface modification and passivation strategies (commonly involving amines, ethylenediamine, polyethyleneimine, polyethylene glycol and thiourea) have been explored to modulate the PL properties with respect to quantum yield (QY), emission wavelength (λ_em_) and fluorescence lifetime (τ) [17,20]. For example, Sun et al. demonstrated that C-dots derived via laser ablation of a carbon target exhibit no fluorescence, but when subjected to acid treatment and subsequent attachment of non-emissive passivation agents such as diamine-terminated oligomeric poly(ethylene glycol) or poly(propionylethylene-imine-co-ethyleneimine), they become strongly fluorescent [21]. Another study demonstrated that biomass-derived C-dots treated with ethanolamine are hydrophilic in nature, but when treated with oleylamine, they are readily dispersible in toluene [22].

With the overarching goal of developing methods for large-scale, low-cost synthesis of photoluminescent C-dots, we propose here a post-synthesis treatment of citric acid (CA)/urea-derived C-dots via NaClO, a strong oxidizing agent known for its capacity to degrade otherwise stable carbon structures. We demonstrate that NaClO-treated C-dots show a six-fold improvement in their PL properties, coupled with advanced antifungal performance, without compromising their non-toxic nature.

## 2. Experimental Section

### 2.1. C-Dot Synthesis

The synthesis of C-dots is based on a protocol previously developed in our laboratory [23]. In brief, a mixture of CA and urea with a molar ratio of CA to urea of f_CA/urea_ = 1:100 was placed on a crucible and heated at 230 °C for 1 h, the product was ground into fine powder, dissolved in water and was purified by dialysis against water using Snakeskin Dialysis Tubing with a Molecular Weight Cut-off (MWCO) of 3.5 kDa for 4 weeks, during which the water was refreshed on a daily basis. Under those experimental conditions, essentially the totality of the unbound small molecules were removed from the retentate, and the dialysate solution did not show any fluorescence emission upon UV illumination. Finally, the product was freeze-dried and kept in a desiccator.

### 2.2. NaClO Treatment

Varying amounts of 0.1 M aqueous solutions of NaClO were added under stirring into beakers containing 20 mL of 0.1 mg/mL aqueous dispersions of C-dots to prepare a series of oxidised C-dots (ox-C-dots), as summarised in Table 1. The mixtures were left at room temperature for 24 h before their PL spectra were recorded. Prior to all other tests, ox-C-dots were subjected to dialysis against water for 4 weeks using Snakeskin Dialysis Tubing with a MWCO = 1 kDa and freeze-dried.

### 2.3. Fourier Transform Infrared (FTIR)

FTIR spectra of freeze-dried C-dots and ox-C-dots were collected within the range of 4000–500 cm^−1^ by means of a Nicolet IS5 spectrometer and the samples were scanned 128 times at a resolution of 2 cm^−1^. Identical amounts were used for all samples studied.

### 2.4. Transmission Electron Microscopy (TEM)

TEM images were captured by means of a Tecnai F20 microscope (FEI Company, Thermo Fisher Scientific, Hillsboro, OR, USA) operated at 200 kV equipped with an eagle camera and TIA software. A drop of a 0.1 mg/mL aqueous dispersion of C-dots was deposited on the carbon-coated copper grid and the solvent was left to evaporate at room temperature, and the same process was followed for ox3-C-dots. The size of the nanoparticles reported here represents the average from 50 readings using a suitable software.

### 2.5. X-Ray Photoelectron Spectroscopy (XPS)

The XPS spectra of the C-dots and ox3-C-dots were collected by a K-alpha spectrometer (Thermo Fisher, Loughborough, UK) equipped with a monochromatic Al Kα X-ray radiation source, and CasaXPS Version 2.3 software was used to fit the data.

### 2.6. Elemental Analysis

Elemental analysis was conducted utilizing the Flash 2000 CHNS-O Analyzer (Thermo Scientific, Loughborough, UK). The instrument was calibrated against 2,5-(Bis(5-tert-butyl-2-benzo-oxazol-2-yl) thiophene (Thermo Scientific, UK). The samples were inserted into aluminium and silver pans for CHN and O analysis, respectively. Measurements were carried out in triplicate and the average values are reported.

### 2.7. Ultraviolet–Visible (UV–Vis) Spectra

Aqueous dispersions of C-dots and ox-C-dots were placed in quartz cuvettes with a 1 cm pathlength, and the spectra were recorded at room temperature using a UV-3600 spectrophotometer (Shimadzu, Torrance, CA, USA).

### 2.8. Photoluminescence

Aqueous dispersions of C-dots and ox-C-dots were placed in quartz cuvettes with a 1 cm pathlength, and their PL spectra were recorded via a Horiba Fluoromax spectrofluorometer (Kyoto, Japan) at excitation wavelengths (λ_ex_) between 300 and 620 nm. Likewise, the QY of aqueous dispersions of C-dots and ox-C-dots was determined against anthracene (Sigma Aldrich, Gillingham, UK) dispersed in ethanol with QY_R_ = 0.27 at λ_ex_ = 365 nm as the reference dye and calculated based on the equation:

QY = QY_R_ × (I/I_R_) × (A_R_/A) × (η^2^/η_R_^2^) (1)

where I refers to the integrated fluorescence intensity of the material in question, A refers to its absorbance value and η is the refractive index of the solvent, while the subscript R denotes the anthracene.

### 2.9. Photoluminescence Lifetime

PL lifetime decays of aqueous dispersions of C-dots and ox-C-dots in quartz cuvettes (1 cm pathlength) were recorded using an Edinburgh Instruments LifeSpec-II (Livingston, UK) equipped with pulsed diode lasers operating at 450 nm (EPL-450) and 375 nm (EPL-375). An aqueous dispersion of silicon dioxide nanoparticles (Ludox HS-30, Sigma Aldrich) was used to calculate the instrument response function. The average PL lifetimes (τ_avg_) were calculated based on the equation:

*τ*_avg_ = ∑*α*_*i*_*τ*_*i*_^2^∑*α*_*i*_*τ*_*i*_ (2)

where *τ*_*i*_ is the time component of multiexponential decay fitting and *α*_*i*_ is the fractional weight for each time component.

### 2.10. Zeta Potential

Zeta potential (ζ) values of 0.05 mg/mL aqueous dispersions of C-dots and ox3-C-dots were measured at 25 °C using a Malvern Panalytical (Malvern, UK) Zetasizer Nano-ZS equipped with a 532 nm He-Ne laser. The pH values were adjusted using 1.0 M HCl and 1.0 M NaOH, the measurements were carried out in triplicate and the average values were calculated.

### 2.11. Cytotoxicity

The cytotoxicity of C-dots and ox3-C-dots against HeLa cervical cancer and U87 glioblastoma cell lines was assessed using MTT (3-(4,5-dimethylthiazolyl-2)-2,5-diphenyltetrazolium bromide) and Prestoblue (based on resazurin) assays (both types of assays were obtained from Sigma Aldrich). The cell culture took place in Dulbecco’s modified Eagle medium (DMEM, Thermo Scientific, UK) that contained 1% penicillin-streptomycin (10,000 U/mL, Thermo Fisher Scientific) and 10% fetal bovine serum (Thermo Fisher Scientific). A total of 50 µL of cell dispersions (1 × 10^5^ cells/mL) were seeded in a 96-well plate and were incubated (the incubation time was overnight for MTT, 10 min for Prestoblue) at 37 °C and 5% CO_2_, before being further incubated with C-dots and ox3-C-dots for another 24 h. Subsequently, 20 µL of a 5 mg/mL MTT solution was transferred into each well and the system was incubated for another 2 h followed by the addition of 150 µL of a 0.7 M lysis buffer into each well. Finally, the optical density (OD) of the dispersions at 595 nm was measured by a microplate reader (Thermo Fisher Scientific). Cell viability was determined via the relationship:

Cell viability (%) = O_Dtreated_/O_Dcontrol_ × 100 (3)

where O_Dcontrol_ and O_Dtreated_ were recorded in the absence and the presence of the nanoparticles, respectively. Measurements were performed three times, and the average values are reported.

### 2.12. Haemolysis Tests

Identical circular wells were created on Columbia Agar with 5% Horse Blood (Scientific Laboratory Supplies, Nottingham, UK), which were filled with 100 μL of a 0.1 mg/mL aqueous dispersions of nanoparticles. We note that ¼ strength Ringer’s solution (Thermo Fisher Scientific) and 1% Triton X-100 in water were used as the negative and positive control, respectively. All samples were incubated for 24 h at 25 °C before the visual observations were made and the photos were captured.

### 2.13. Antifungal Activity

*Talaromyces pinophilus* (*T. pinophilus*) ATCC 11797 was obtained from the American Type Culture Collection (ATCC) and maintained on potato dextrose agar (PDA) plates, stored within a refrigerated environment maintained between 3 to 5 °C. For the preparation phase, 10 mL of sterile distilled water was introduced into a 50 mL sterile falcon tube. A plate of *T. pinophilus* was carefully transferred into the tube, where it was subjected to a thorough grinding process to liberate the spores. The resulting mixture was then meticulously filtered through sterile filter floss to remove any residual debris. Following filtration, the spore-containing suspension underwent centrifugation at 4000 rpm for 5 min, leading to the separation of the spores from the supernatant. This supernatant was discarded, while the concentrated spores were resuspended in another 10 mL of sterile distilled water. This suspension was subjected to a second round of centrifugation to further purify the spores, with the supernatant once again being discarded. The purified spores were then resuspended in a nutrient salt solution and adjusted to 1,000,000 ± 200,000 spores/mL with the aid of a haemocytometer. The nutrient salt solution was prepared by dissolving 1.0 g ammonium sulfate, 0.7 g dipotassium phosphate, 0.7 g monopotassium phosphate, 0.7 g magnesium sulfate, 0.005 g sodium chloride, 0.002 g ferrous sulfate, 0.002 g zinc sulfate and 0.001 g manganous sulfate in 1 L of distilled water. Sterile polymer samples, cut to approximately 10 mm × 10 mm or 25 mm × 25 mm in size, were soaked in either water (serving as the control) or 0.1 mg/mL C-dots solutions for a period of 30 min.

### 2.14. Design of Polymer Composites

The polymers considered in this study are: a polyvinylidene fluoride (PVDF) transfer membrane (porosity 0.45 μm) from Thermofisher, a standard kitchen polyurethane sponge and a polyester fabric from Sanil industries. Following soaking, the samples were removed and allowed to dry completely. Subsequently, 100 μL of the fungal spore suspension was taken and a portion spread evenly onto PDA plates. Control and test squares were placed onto the surface of the plates, one square per plate, and the remaining portion of the spore suspension was added atop the sample. A thin layer of PDA of approximately 45 °C was then poured onto each plate, ensuring both the polymer square and pre-existing agar were covered completely. Plates were allowed to dry before being photographed, then incubated at 25 °C for 5 days. Following incubation, the plates were visually assessed and photographed again. All tests were carried out in duplicate.

## 3. Results and Discussion

**Figure 1:**
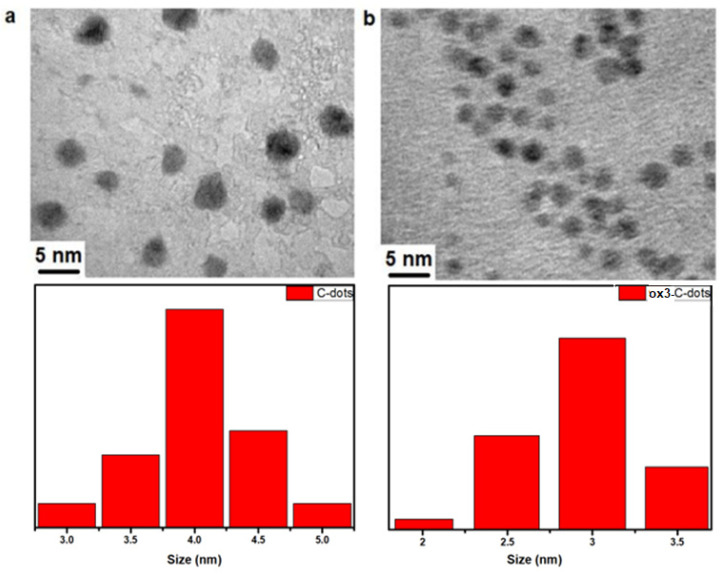
TEM images and corresponding size distribution histograms (n = 50) of (**a**) C-dots and (**b**) ox3-C-dots.

As discussed in the experimental section, the C-dots were retained within the dialysis membrane with MWCO = 3.5 kDa (Supplementary Figure S1a), in sharp contrast to ox-C-dots that permeated the pores (Supplementary Figure S1b), thus necessitating the use of a membrane with MWCO = 1 kDa for their effective purification (Supplementary Figure S1c). TEM imaging (Figure 1) revealed the presence of spherical nanoparticles, with an average size of 4.3 nm and 2.9 nm for C-dots and ox3-C-dots, respectively.

Elemental analysis (Supplementary Table S1) indicated a substantial increase in the stoichiometric amount of oxygen as a result of the NaClO treatment from 25.6% for C-dots to 38.2% and 49.5% for ox3-Cdot and ox7-C-dots, respectively. At the same time, the carbon percentage remained within the range of 38–43% for all samples considered, while the nitrogen content was found to decrease from 32.4% for C-dots to 17.6% and 11.9% for ox3-Cdots and ox7-C-dots, respectively. Analysis of XPS survey spectra indicated the presence of 41.6% C1s, 35.5% N1s and 22.9% O1s for C-dots, compared to 40.3% C1s, 19.9% N1s and 39.7% O1s for ox3-C-dots. These data further confirm the substantial decrease in nitrogen content coupled with the pronounced increase in oxygen content as a direct consequence of the NaClO treatment. It is well-established that NaClO can react with a variety of nitrogen compounds to form volatile chloramines and nitrogen trichloride [24], and this behaviour might account for the substantial decrease in nitrogen content observed for ox-C-dots in this study. We note that colourless NaClO-oxidised C-dots dispersions instantly turned into white turbid solutions when CaCl_2_ was added, a behavior consistent with the presence of carbonated ions due to the formation of CO_2_ as a decomposition product, in line with previous studies [25].

**Figure 2:**
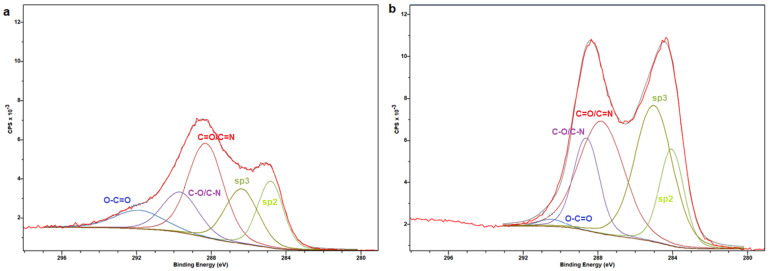
Deconvolution of C1s XPS spectra of (**a**) C-dots and (**b**) ox3-C-dots.

The C1s XPS spectrum of C-dots (Figure 2) can be deconvoluted into 18.5% sp^2^, 19.7% sp^3^, 36.1% C=O/C=N, 15.8% C-O/C-N and 9.8% O-C=O, while the corresponding spectrum for ox3-C-dots suggests the presence of 15.6% sp^2^, 34.1% sp^3^, 33.3% C=O/C=N, 15.8% C-O/C-N and 1.2% O-C=O. Data derived from C1s, O1s, N1s XPS analysis are summarised in Supplementary Tables S2, S3 and S4, respectively.

**Figure 3:**
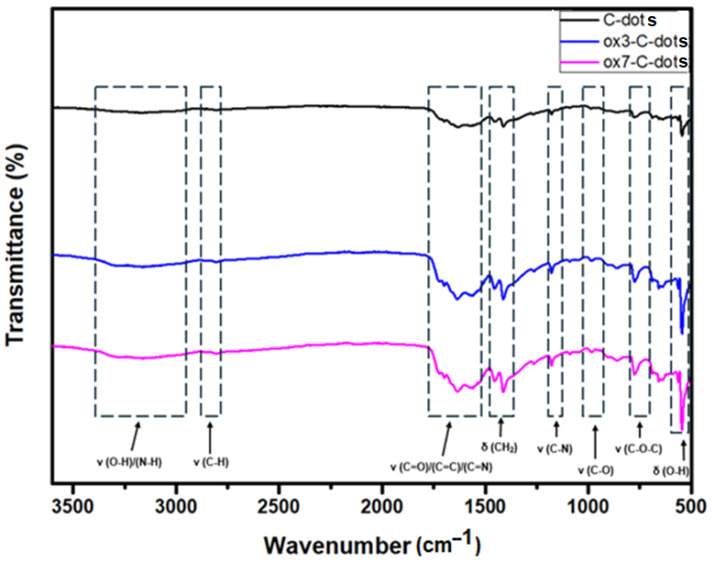
FTIR spectra of C-dots, ox3-C-dots and ox7-C-dots.

The FTIR spectrum of C-dots (Figure 3) is dominated by peaks centred at 550 cm^−1^ (bending of O-H), 851 cm^−1^ (stretching of C-O-C), 967 cm^−1^ (anti-symmetrical stretching of C-O), at 1175 cm^−1^ (stretching of C-N), 1463 cm^−1^ (bending of CH_2_ groups), 1554 cm^−1^–1723 cm^−1^ (stretching of bonds involving C=C, C=O, and C=N) and 2869 cm^−1^ (stretching of C-H) [26,27]. In general lines, the FTIR peaks of ox3-C-dots and ox7-C-dots do not exhibit displacement compared to C-dots, but their intensity appears much stronger, indicating an increased population of the functional surface groups.

Taken together, the data presented above point to extensive oxidation effects on the C-dots’ surfaces induced by NaClO that result in major structural rearrangements, analogous to the UV-assisted degradation of GO by NaClO previously reported that proceeds via skeleton carbon cleavage and attack of the epoxy and alkoxy units to generate -COOH peripheral groups [28]. By virtue of its strong oxidizing nature, NaClO is able to break C=C bonds and C=N imine groups while attacking hydroxyl groups and ether bonds [29,30], ultimately degrading single-walled carbon nanotubes (SWCNT), multi-walled carbon nanotube, nanohorns and graphene oxide [25,31,32]. This behaviour is distinctly different compared to KIO_4_, KMnO_4_ and K_2_Cr_2_O_7_, which are able to oxidize hydroxyl groups on the C-dots’ surfaces, but do not cause structural decomposition [33].

**Figure 4:**
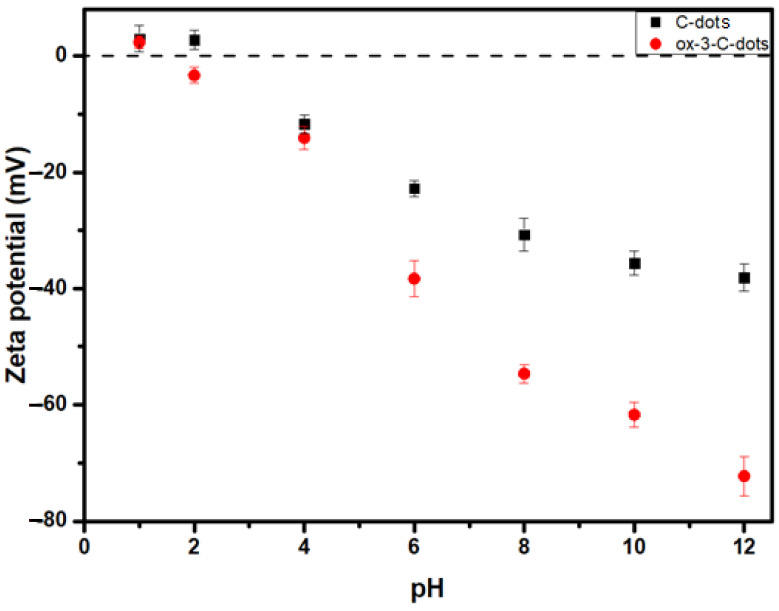
Zeta potential of 0.05 mg/mL aqueous dispersions of C-dots (black squares) and ox-3-C-dots (red circles).

As shown in Figure 4, C-dots show slightly positive ζ values for pH values of 1 and 2, but adopt negative values in less acidic environments, showing ζ = −38 mV at pH = 12 due to the deprotonation of surface hydroxyl and carboxyl groups [34]. At the same time, ox3-C-dots show small positive values ζ = 2 mV at pH = 1, but at higher pH values ζ becomes negative, reaching ζ = −73 mV at pH = 12. This behaviour points to an increased population of oxygenated functional groups on the ox3-C-dots and suggests an enhanced colloidal stability as a result of the surface treatment.

**Figure 5:**
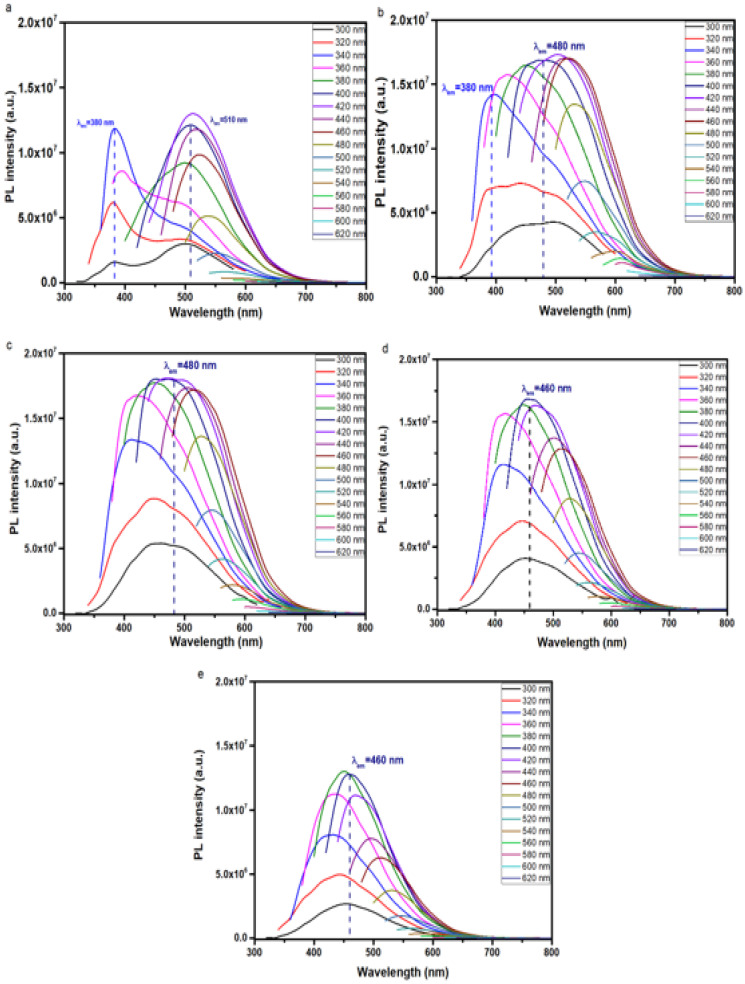
PL spectra of 0.1 mg/mL aqueous dispersions of (**a**) C-dots, (**b**) ox1-C-dots, (**c**) ox2-C-dots, (**d**) ox3-C-dots and (**e**) ox7-C-dots at λ_ex_ indicated.

The PL spectra of aqueous dispersions of C-dots (Figure 5a) follow the characteristic λ_ex_-dependent emission mode at λ_ex_ > 380 nm and the λ_em_ redshifts upon increasing λ_ex_, as expected for this class of materials. The occurrence of organic fluorophores well-embedded within the carbogenic core, or the nanoparticle peripheral, is manifested by the presence of distinct λ_ex_-independent contributions observed at λ_em_ = 380 nm and 510 nm (Figure 5a), corresponding to the blue dye citrazinic acid (CTA) and green dye 4-hydroxy-1H-pyrrolo [3,4-c]pyridine-1,3,6(2H,5H)-trione (HPPT), respectively [35]. The UV–vis spectrum of C-dots (Supplementary Figure S2) displays an absorption peak at 275 nm that is associated with the π-π* transitions of the pyridone ring of HPPT, while the peaks at 335 and 415 nm are typical for aromatic systems and indicate the formation of CTA [23,35]. The absorption peaks and λ_ex_-independent PL contributions observed in C-dots can be discerned for ox1-C-dots (Figure 5b), but are diminished for ox2-C-dots, ox3-C-dots and ox7-C-dots (Figure 5c–e, respectively), indicating the gradual degradation of organic fluorophores upon the addition of NaClO. This observation is in agreement with previous studies that demonstrated the capacity of NaClO to break down organic dyes [36,37]. 

**Figure 6:**
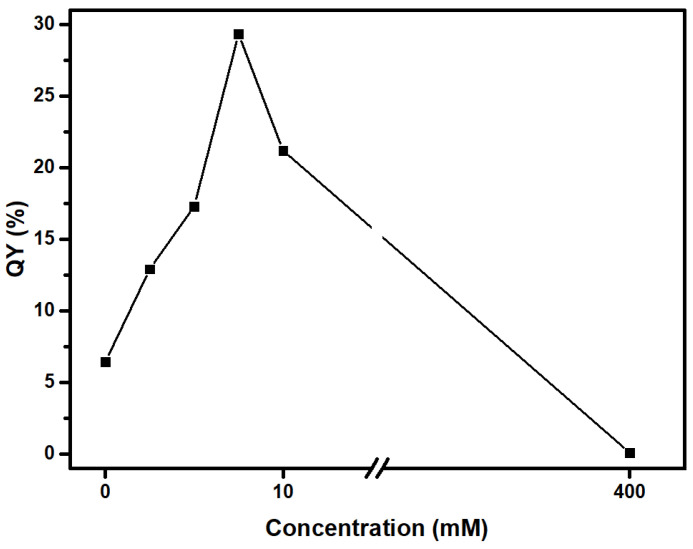
QY of C-dots and ox-C-dots as a function of the concentration of NaClO used for the treatment of C-dots as detailed in Table 1.

As shown in Figure 6, the QY (λ_ex_ = 365 nm) was found to be close to 6.5% for C-dots, increases to 12.9%, 17.3% and 29.4% for ox1-C-dots, ox2-C-dots and ox3-C-dots, respectively, and then falls to 21.2% for ox4-C-dots. At the same time, QY approaches zero for ox8-C-dots, pointing to pronounced decomposition of the dispersed nanoparticles in the presence of excessive amounts of NaClO.

In other words, the oxidative treatment presented here, on the one hand, diminishes the PL contributions associated with the presence of organic fluorophores, but, on the other hand, the PL emissions stemming from the carbogenic core and the peripheral groups are substantially enhanced up to a certain concentration of added NaClO. Interestingly, the emission peaks (λ_ex_ = 460 nm) for C-dots and ox1-C-dots can be fitted to one Gaussian curve (Supplementary Figure S3a,b), but the corresponding peak for ox2-C-dots, ox3-C-dots and ox7-C-dots can be deconvoluted into two Gaussian curves (Supplementary Figure S3c–e, respectively). In each sample, the narrow P1 curve is associated with the intrinsic emission from the core, while the broad P2 curve arises from the extrinsic emission shell emissions [38]. It can be seen that P2 curves become progressively more intense as the amount of NaClO increases, indicating a major structural organisation of C-dots induced by NaClO.

**Figure 7:**
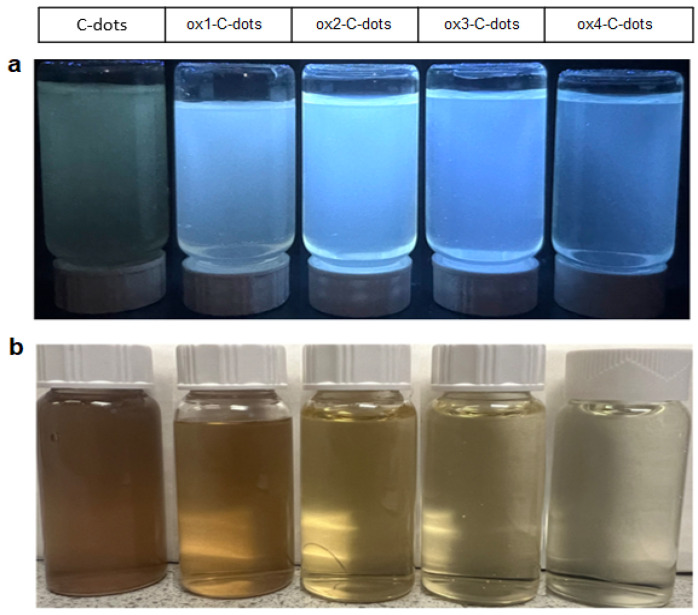
Photos of aqueous dispersions of ox-C-dots under (**a**) UV radiation and (**b**) daylight compared to the original (untreated) C-dot dispersion. All photos were taken 24 h following the addition of NaClO.

The photos of C-dots and ox-C-dots under UV illumination (Figure 7, upper photos) demonstrate the stronger PL signals emitted by ox-C-dots compared to C-dots. Note that C-dots appear turquoise, while ox-C-dots appear light blue, an observation consistent with the decomposition of the green dye HPPT. At the same time, the photos captured under daylight (Figure 7, lower images) suggest that the brown colour of C-dots gradually weakens upon addition of NaClO, in line with the reduced absorbance within the visible area of the spectrum shown in Supplementary Figure S2.

For reference, it is noted that catalytically induced surface oxidation over a period of 24 h on sucrose derived C-dots (N-methyl morpholine N-oxide was used as the catalyst) resulted in a six-fold increase in QY [39], while glucose-derived C-dots that underwent aerial oxidation in the presence of MgSO_4_ over a period of 6 months demonstrated an increase in QY from 0.61% to 4.26% [40]. Furthermore, electrogenerated hypochlorite ions were seen to enhance the QY of CA/ethanolamine-derived C-dots up to 640% [41].

The PL relaxation dynamics at λ_ex_ = 375 nm reveal that τ_avg_ is close to 2.9 ns, 2.8 ns, 2.7 ns and 1.9 ns for C-dots, ox2-C-dots, ox4-C-dots and ox7-C-dots, respectively (Supplementary Figure S4), while at λ_ex_ = 450 nm, the τ_avg_ values were found to be 7.2 ns, 5.7 ns, 4.3 ns and 3.7 ns, respectively; the fitting parameters are listed in Supplementary Table S5. The multi-exponential PL delay mechanisms typically observed for this class of materials is believed to originate from heterogeneous emitting states found within individual nanoparticles and their larger ensembles. The fast delay mechanisms have been attributed to the recombination of intrinsic states, while the slower contributions have been attributed to defect states [42].

**Figure 8:**
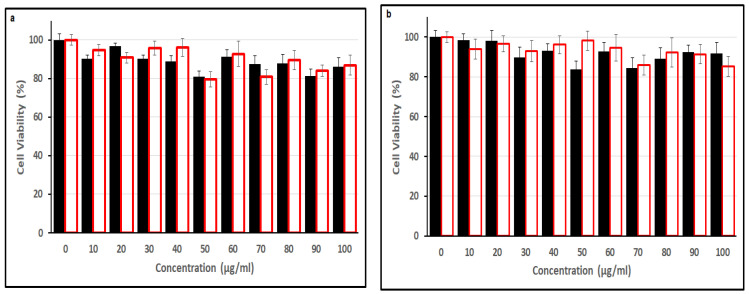
MTT assay test to assess the viability of HeLa (**a**) and U87 cells (**b**) following their 24 h incubation with C-dots (black bars) and ox3-C-dots (red bars).

MTT and Prestoblue assays (Figure 8 and Supplementary Figure S5, respectively) were used to assess the cytotoxicity of C-dots and ox3-C-dots. The viability of Hela and U87 cells incubated for 24 h with 100 µg/mL (the highest concentration considered) of C-dots and ox3-C-dots, respectively, remained above 90%, while similar results were obtained using Prestoblue assays. In addition, haemolysis tests (Supplementary Figure S6) in the presence of 0.1 mg/mL C-dots and ox3-C-dots suggested γ haemolysis (no haemolysis), in line with previous studies in closely related systems [43,44]. Previous studies demonstrated the pivotal role of the surface chemistry in determining the cellular association, cytotoxic activity and trafficking of nanoparticles [45].

**Figure 9:**
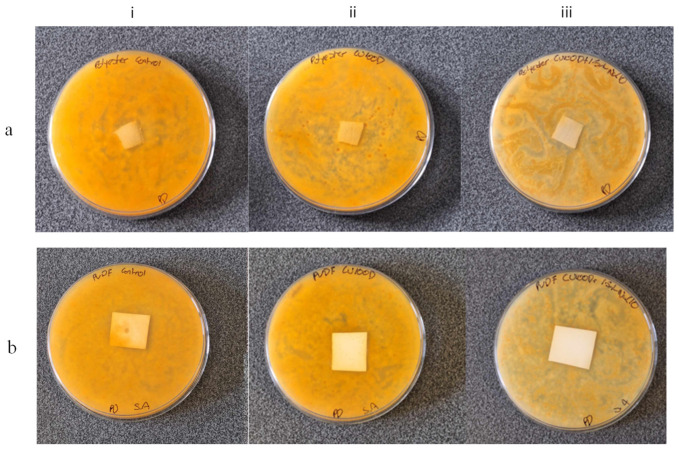
Photos of the Petri dishes containing *T. Pinophilus* cultures in the presence of polyester fabric (**a**) and PVDF membrane (**b**) impregnated with (i) water (control), (ii) C-dots and (iii) ox3-C-dots.

Figure 9a displays photos of the agar plates containing *T. Pinophilus* cultures in the presence of polyester fabric impregnated with water (i) (control test), C-dots (ii) and ox3-C-dots (iii). It is apparent that, compared to C-dots, ox3-C-dots are more effective in combatting fungal growth given that they create large zones of inhibition with swirling motifs that spread across the entire plate, mirroring the pathways of diffusion of the nanoparticles. Likewise, the advanced antifungal activity of ox3-C-dots-impregnated PVDF membranes is evident from the lighter yellow colour of the corresponding agar plate compared to the control, while intermediate colour tones are seen for C-dots plates (Figure 9b). Similar trends were observed for polyurethane sponges impregnated with C-dots and ox3-C-dots (Supplementary Figure S7). The antifungal activity modes of C-dots have been attributed to their selective interactions with ergosterol and their capacity to trigger uncontrolled production of reactive oxygen species (ROS), leading to DNA damage and protein denaturation [46]. We note that ROS production is more pronounced in the presence of hydrogen-donating groups (such as carbonyl and hydroxyl groups) [47], an effect which might be responsible for the higher levels of antifungal activity observed for ox3-C-dots.

Taken together, all the data reported here indicate the generation of oxygenated functional groups on the C-dots’ surfaces and the disintegration of organic fluorophores during the early NaClO treatment stages, followed by nanoparticle etching and shrinkage due to pronounced surface oxidation and the release of CO_2_ at intermediate stages, ultimately leading to complete decomposition of NPs. During this process, the QY is seen to first increase rapidly, despite the removal of fluorophores, due to enhanced emissions associated with the carbogenic core and its periphery, and then decrease due to the gradual reduction in the number of nanoemitters.

## 4. Conclusions

We demonstrate here that direct NaClO treatment of C-dots results in pronounced surface oxidation and etching, a process that facilitates release of CO_2_. This treatment simultaneously suppresses UV-vis absorbance, diminishes the fluorescence emission stemming from organic fluorophores, increases the QY by a factor of six and substantially enhances the antifungal activity. This approach offers a time- and cost-effective strategy to modulate the size, optical and antimicrobial properties of C-dots, without compromising their non-toxic nature.

## Figures and Tables

**Table 1:** Sample description of ox-C-dots with respect to the amounts of C-dots and NaClO used for their preparation.

Sample Description	Volume (in mL) of 0.1 mg/mL Dispersion of C-Dots	Volume (in mL) of 0.1 M Solution of NaClO	NaClO Concentration (mM)
οx1-C-dots	20	0.5	2.5
οx2-C-dots	20	1.0	5
ox3-C-dots	20	1.5	7.5
ox4-C-dots	20	2.0	10
ox5-C-dots	20	2.5	12.5
ox6-C-dots	20	3.0	15
ox7-C-dots	20	4.0	20
ox8-C-dots	20	2.0 *	400

* Concentration 4.4 M.

## Data Availability

Data are contained within the article.

## Supplementary Materials

The following are available online at https://www.mdpi.com/article/10.3390/nano15030184/s1, Supplementary Figure S1. Photos depicting dialysis experiments of C-dots under illumination at λ_ex_=365 nm. (a) C-dots in a 3.5 kDa MWCO membrane, (b) ox3-C-dots in 3.5 kDa MWCO membrane and (c) ox3-C-dots in 1 kDa MWCO membranes (Photos were captured 48 h following the commencement of dialysis), Supplementary Figure S2. UV-vis spectra of aqueous dispersions containing 0.1 mg/ml C-dots, ox1-C-dots, ox2-C-dots, ox3-C-dots, ox-4-C-dots, respectively, Supplementary Figure S3. Fitted emission peaks (λ_ex_ =460 nm) of 0.1 mg/ml aqueous dispersions of (a) C-dots (b) ox1-C-dots, (c) ox2-C-dots, (d) ox3-C-dots and (e) ox7-C-dots, Supplementary Figure S4. PL lifetime decays of aqueous dispersions containing 0.1 mg/ml C-dots, ox3-C-dots and ox7-C-dots, respectively at (a) λ_ex_=375 nm and (b) 450 nm, Supplementary Figure S5. Viability of HeLa and U87 cells (based on Prestoblue assay) after being incubated for 24 h with C-dots and ox3-C-dots, Supplementary Figure S6. Haemolysis tests showing gamma (no) haemolysis induced by the presence of 0.1 mg/mL aqueous dispersion of C-dots, ox3-C-dots and the negative control as opposed to beta (complete) haemolysis shown for the positive control, Supplementary Figure S7. Photos of the Petri dishes containing *T. Pinophilus* cultures in the presence of polyurethane sponge impregnated with (i) water (control), (ii) C-dots, (iii) ox3-C-dots, Supplementary Table S1. Elemental analysis of C-dots, ox3-C-dots and ox7-C-dots, Supplementary Table S2. Data derived from the deconvolution of C1s XPS spectrum of C-dots and ox3-C-dots, Supplementary Table S3. Data derived from the deconvolution of O 1s XPS spectrum of C-dots and ox3-C-dots, Supplementary Table S4. Data derived from the deconvolution of N 1s XPS spectrum of C-dots and ox3-C-dots, Supplementary Table S5. PL lifetime fitting parameters of C-dots, ox2-C-dots, ox4-C-dots and ox7-C-dots.
