# Supplementary material for: Enhancing the Fluorescence and Antimicrobial Performance of Carbon Dots via Hypochlorite Treatment
Source: Nanomaterials (Basel). 2025 Jan 24;15(3):184. doi: 10.3390/nano15030184 (PMC11819752; doi:10.3390/nano15030184)
Supplement: Supplementary file 1 [file nanomaterials-15-00184-s001.zip › nanomaterials-3422627-supplementary.pdf]

Supplementary Information

Enhancing the fluorescence and antimicrobial performance of Carbon dots via hypochlorite treatment

Spyridon Gavalas, Mohammed S. Beg, Ella Gibbons, Antonios Kelarakis*

UCLan Research Centre for Smart Materials; School of Pharmacy and Biomedical Sciences, University of Central Lancashire, Preston PR12HE, UK.

* Correspondence: akelarakis@uclan.ac.uk; Tel.: 004417724172

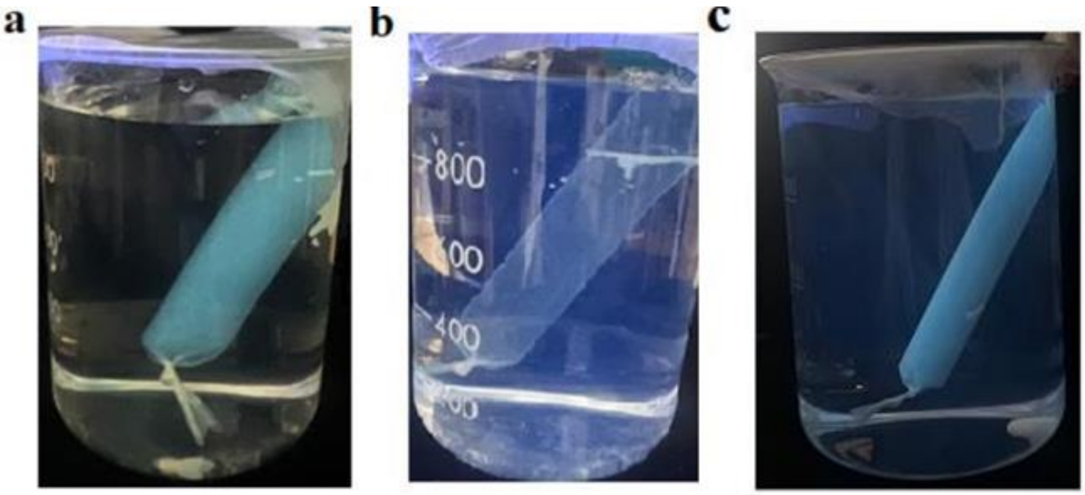

Supplementary Figure S1. Photos depicting dialysis experiments of C-dots under illumination at λ_{ex}=365 nm. (a) C-dots in a 3.5 kDa MWCO membrane, (b) ox3-C-dots in 3.5 kDa MWCO membrane and (c) ox3-C-dots in 1 kDa MWCO membranes (Photos were captured 48 h following the commencement of dialysis).

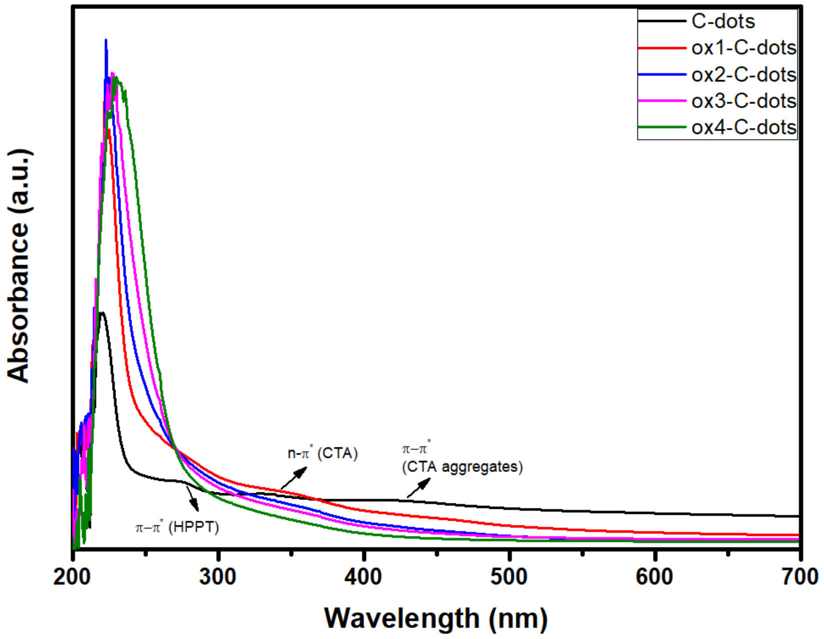

Supplementary Figure S2. UV-vis spectra of aqueous dispersions containing 0.1 mg/ml C-dots, ox1-C-dots, ox2-C-dots, ox3-C-dots, ox-4-C-dots, respectively.

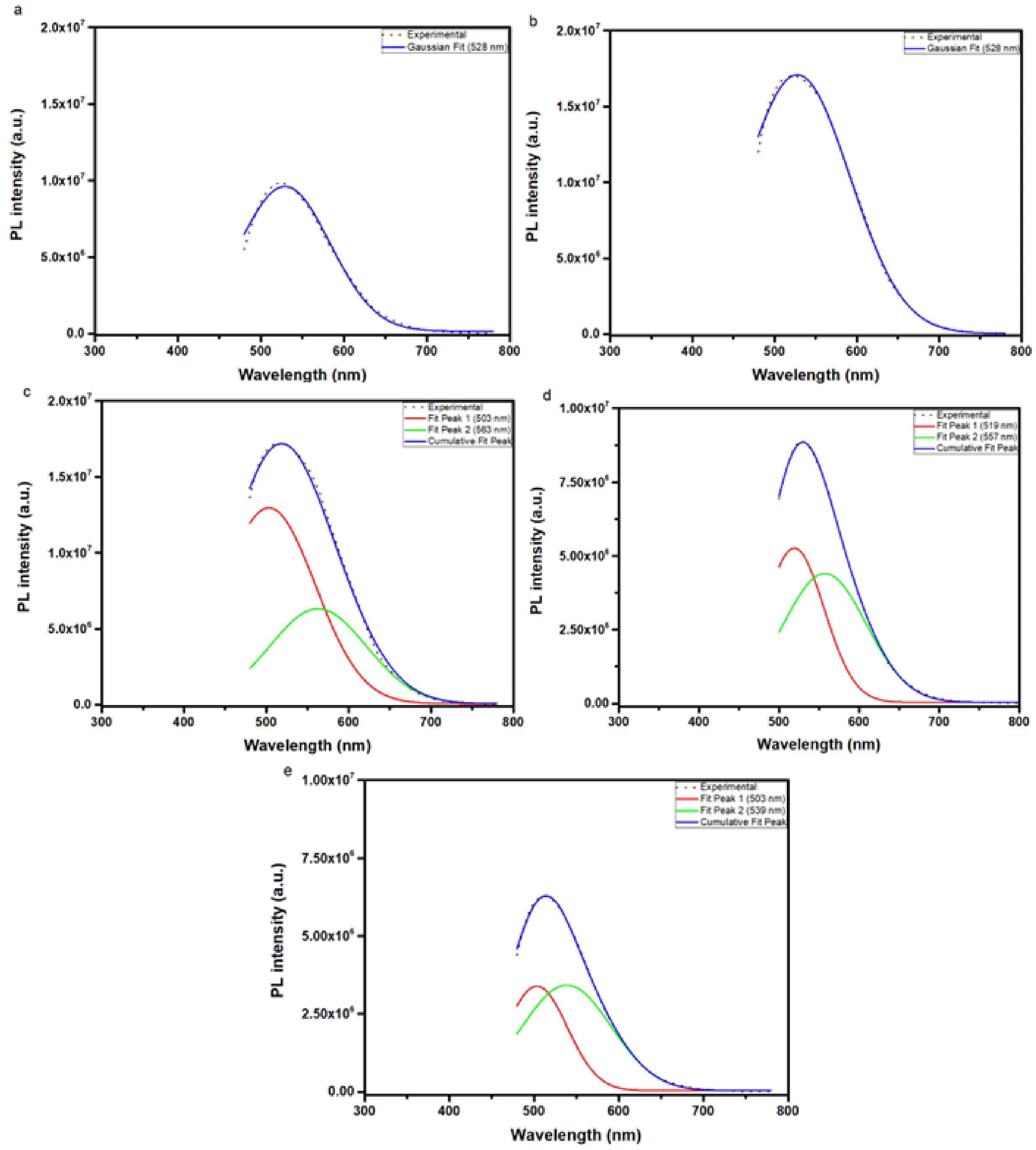

Supplementary Figure S3. Fitted emission peaks (λ_{ex} =460 nm) of 0.1 mg/ml aqueous dispersions of (a) C-dots (b) ox1-C-dots, (c) ox2-C-dots, (d) ox3-C-dots and (e) ox7-C-dots.

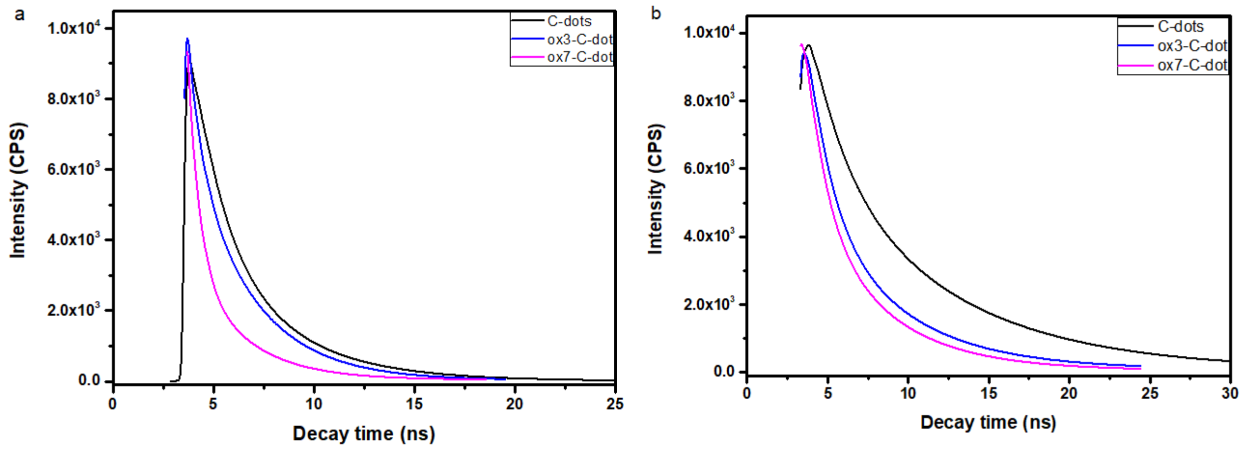

Supplementary Figure S4. PL lifetime decays of aqueous dispersions containing 0.1 mg/ml C-dots, ox3-C-dots and ox7-C-dots, respectively at (a) λ_{ex}=375 nm and (b) 450 nm.

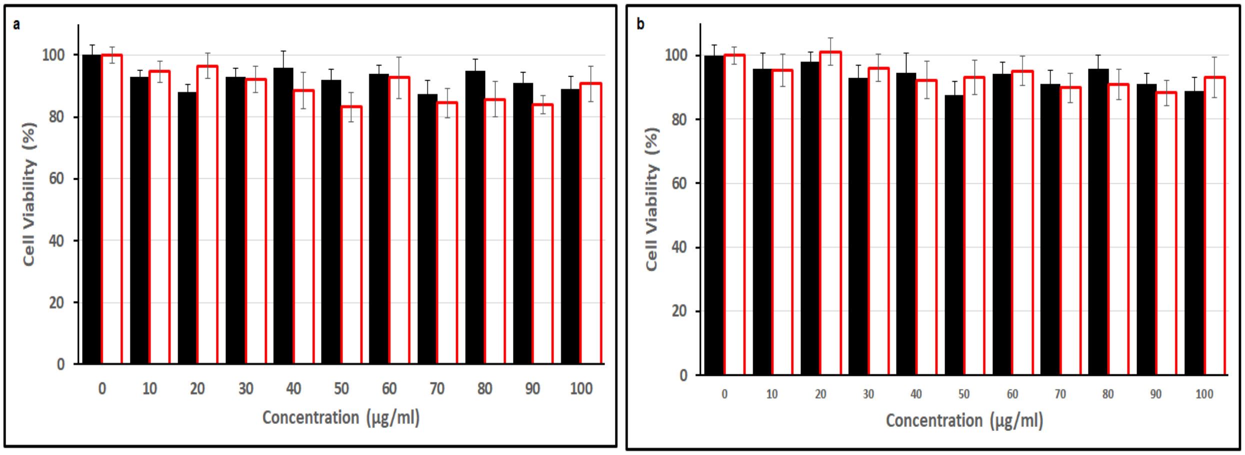

Supplementary Figure S5. Viability of (left) HeLa and (right) U87 cells (based on Prestoblue assay) after being incubated for 24 h with C-dots and ox3-C-dots.

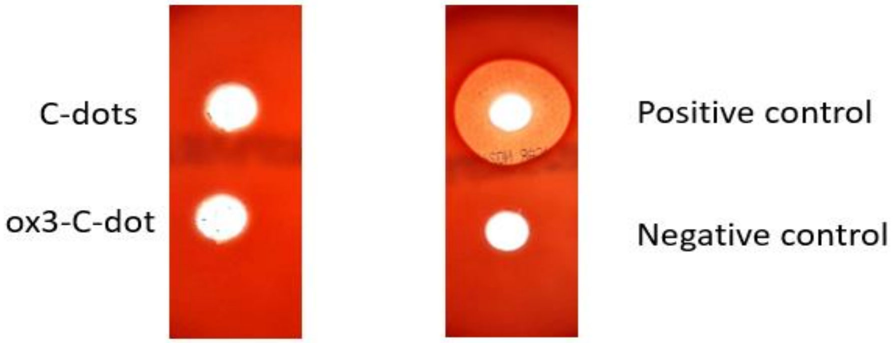

Supplementary Figure S6. Haemolysis tests showing gamma (no) haemolysis induced by the presence of 0.1 mg/mL aqueous dispersion of C-dots (left upper), ox3-C-dots (left lower) and the negative control (right lower) as opposed to beta (complete) haemolysis shown for the positive control (right upper).

i ii iii

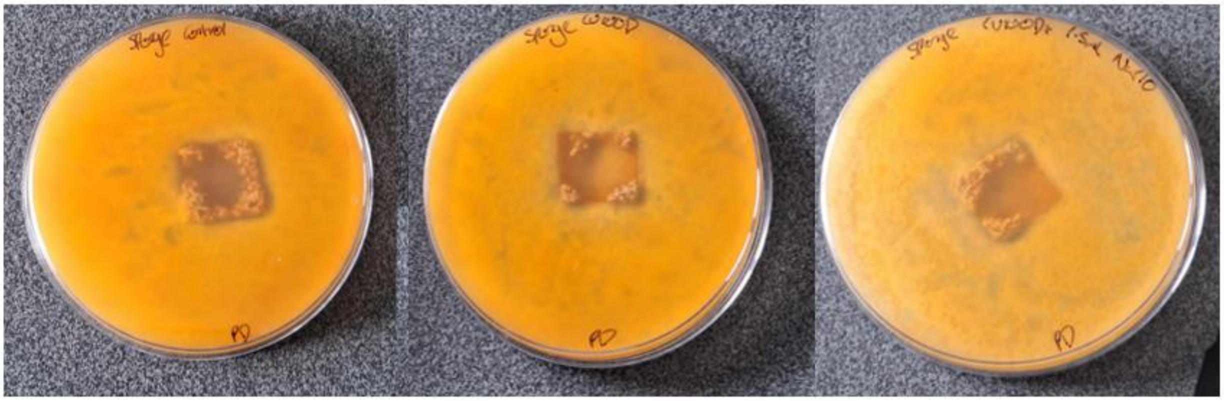

Supplementary Figure S7. Photos of the Petri dishes containing *T. Pinophilus* cultures in the presence of polyurethane sponge impregnated with i) water (control), ii) C-dots, iii) ox3-C-dots.

Supplementary Table S1. Elemental analysis of C-dots, ox3-C-dots and ox7-C-dots.

Sample	C %	H %	N %	O %
**C-dot**	38.6	3.4	32.4	25.6
**ox3-C-dot**	43.1	1.1	17.6	38.2
**ox7-C-dot**	37.9	0.7	11.9	49.5

Supplementary Table S2. Data derived from the deconvolution of C1s XPS spectrum of C-dots and ox3-C-dots.

Sample	sp^2 %	sp^3 %	C=O/C=N %	O-C=O %	C-O/C-N %
**C-dots**	18.54	19.71	36.11	9.82	15.83
**ox3-C-dots**	15.64	34.05	33.30	1.22	15.79

Supplementary Table S3. Data derived from the deconvolution of O 1s XPS spectrum of C-dots and ox3-C-dots.

Sample	C=O %	C-O %
**C-dots**	70.16	29.84
**ox3-C-dots**	61.92	38.08

Supplementary Table S4. Data derived from the deconvolution of N 1s XPS spectrum of C-dots and ox3-C-dots.

Sample	Pyrrolic-N %	Pyridinic-N%	Graphitic-N %	Oxidized-N %
**C-dots**	35.44	18.90	29.80	15.87
**ox3-C-dots**	46.60	30.15	23.25	-

Supplementary Table S5. PL lifetime fitting parameters of C-dots, ox2-C-dots, ox4-C-dots and ox7-C-dots.

Sample	Excitation Wavelength (nm)	Time Coefficient τ_1 (ns)	Probability α_1	Time Coefficient τ_2 (ns)	Probability α_2	T_{avg} (ns)
**C-dots**	375	0.615	0.0908	3.1445	0.9092	2.9
	450	2.0079	0.1425	8.0725	0.8575	7.2
**ox2-C-dots**	375	0.5841	0.0971	3.0703	0.9029	2.8
	450	1.9951	0.2671	6.9969	0.7329	5.7
**ox4-C-dots**	375	0.4831	0.1219	2.9714	0.8781	2.7
	450	1.3329	0.2306	5.2217	0.7694	4.3
**ox7-C-dots**	375	0.4687	0.3533	2.6011	0.6467	1.9
	450	1.1174	0.244	4.5207	0.756	3.7
